# Research and Learning Priorities for a Surgical Obstetrics and Family Planning Project Implementing in Low- and Middle-Income Countries: Results of an Expert Consultation

**DOI:** 10.9745/GHSP-D-24-00174

**Published:** 2025-12-31

**Authors:** Farhad A. Khan, Karen Levin, Renae Stafford, Vandana Tripathi

**Affiliations:** a Formerly with MOMENTUM Safe Surgery in Family Planning and Obstetrics, EngenderHealth, Washington, DC, USA.

## Abstract

**Introduction:** Cesarean delivery, peripartum hysterectomy, female genital fistula treatment, and long-acting and permanent contraceptive method provision comprise an important set of surgical procedures in reproductive and maternal health. The volume of these procedures is growing in low- and middle-income countries (LMICs). Establishing research priorities in a learning agenda for surgical obstetrics and family planning represents a key step in generating and using evidence to improve health outcomes associated with these surgeries.

**Methods:** Between January and February 2022, a safe surgery project addressing family planning and obstetrics used a 2-stage rating and ranking consultation process to prioritize topics in its learning agenda, focusing on LMIC needs. A list of research and learning topics spanning the project’s technical areas, consisting of surgical obstetric care (cesarean delivery and peripartum hysterectomy), fistula prevention and treatment, family planning, and cross-cutting safe surgery, was curated by searching the literature, conducting project-related surveys of experts and partners, and soliciting an expert panel via virtual consultation. Through an online survey, the experts rated the 63 topics on a 5-point scale based on 4 criteria—feasibility, technical importance, level of saturation, and potential for impact—and average ratings were calculated for each criterion and topic. The expert panel then reconvened virtually to rank and refine highly rated topics.

**Results:** A total of 39 people participated in the expert panel, representing multilateral, academic, and funding organizations, implementing partners, and professional associations active in LMICs. Fifteen topics were prioritized across the 4 technical areas. Prioritized topics covered themes of prevention (e.g., intrapartum/midwifery practices to prevent unnecessary cesarean delivery), care-seeking (e.g., social and behavior change strategies for fistula prevention), perioperative care (e.g., use of quality improvement tools including checklists and audits), and postoperative care (e.g., effective measurement approaches for monitoring outcomes).

**Conclusion:** This agenda guides clinical and programmatic learning across the safe surgery ecosystem. Collaborative action across program initiatives and clinical and community settings may contribute to significant evidence building in these priority topics.

## Abstract (precis)

We identified 15 research and learning priorities for surgical obstetrics and family planning for a project’s learning agenda through an expert consultation. Practitioners, program implementers, donors, and researchers involved in global safe surgery, maternal health, and family planning can apply these priorities toward guiding their evidence generation and translation efforts.

## Teaser

**Key Finding:**

- Strengthening the provision of safe surgery in low- and middle-income countries is essential for reducing maternal morbidity and mortality; an expert consultation representing international organizations and donors and African universities collaboratively identified 15 research and learning priorities for obstetric care, fistula prevention and treatment, long-acting and permanent contraceptive methods, and cross-cutting safe surgery, covering preventative, care-seeking, perioperative, and postoperative themes.

**Key Implications:**

- Although the topics prioritized in this research and learning agenda were developed in the context of a project, they provide donors in maternal health and family planning with lines of inquiry to target investments in research and learning.
- Practitioners, program implementers, and researchers in surgical maternal health and family planning care can fill evidence gaps by applying these lines of inquiry within their interventions and sharing learning across programs and settings.
- This agenda, in focusing on cross-cutting knowledge gaps related to safe surgery, can complement priority learning questions in national strategic plans for surgery, family planning, and/or maternal health; priorities for policy research; and broader maternal health and family planning research agendas.

## INTRODUCTION

Despite significant reductions over time, the global maternal mortality ratio remains high at 211 deaths per 100,000 live births.^1^ Sustained, accelerated reductions in maternal morbidity and mortality in low- and middle-income countries (LMICs) require universal access to quality, safe surgical services,^2^ including long-acting and reversible contraceptives (LARCs) and permanent family planning methods, cesarean delivery, peripartum hysterectomy, and fistula prevention and treatment. Although significant gaps in global access to safe surgical care exist,^3^ rural facilities often experience limited human and material resources whereas urban facilities typically experience excessive demand for services.^4^ Under both circumstances, facility readiness to perform quality, indicated surgical procedures remains an issue. These issues are particularly important in all settings as the volume of surgical procedures increases without comparable increases in capacity.^5^ These trends are concerning; an example of the consequences is the increasing iatrogenic fistula associated with cesarean delivery and hysterectomy in a number of LMICs.^6^^,^^7^

MOMENTUM Safe Surgery in Family Planning and Obstetrics was the United States Agency for International Development’s (USAID) flagship global safe surgery initiative. The project sought to reduce maternal morbidity and mortality by supporting country health systems actors (governments, institutions, organizations) to strengthen their capacity to provide surgical services, focusing on long-acting and permanent family planning methods, cesarean delivery, peripartum hysterectomy, and fistula prevention and treatment.^8^ As part of the project aims to utilize evidence and adaptive learning methods, identifying knowledge gaps was considered a key step to strengthening surgical systems, and in turn, access and quality.

A learning (or research) agenda comprises questions or topics aiming to address knowledge gaps pertaining to program implementation, activities designed to answer them, and resulting dissemination products.^9^ A strategically developed and implemented learning agenda can accelerate the translation of evidence into practice. For example, in a previous global project,^10^ the prioritization of iatrogenic fistula in that learning agenda was followed by incorporating etiology tracking in routine monitoring systems,^6^^,^^11^ provider surveys, a technical consultation on cesarean delivery safety,^12^ and ultimately a body of evidence and action contributing to focus on surgical safety. An association between gender-based violence (GBV) and fistula was observed in a study that was done in response to the need to characterize sociocultural factors of fistula,^13^ which is currently being translated into practice by means of integrating GBV screening and referral into treatment.

Current research agendas on safe surgery in LMICs focus on areas of unmet need for surgical patients^14^ and knowledge gaps needed for surgical service delivery and policy in Southern Africa.^15^ Disseminated research priorities in maternal, newborn, and child health and reproductive health have included fistula prevention and treatment;^11^ sexual and reproductive health and rights (SRHR) for the World Health Organization (WHO) African Region;^16^ family planning in Uganda,^17^ Niger,^18^ and Mozambique;^19^ and improving respectful care for newborns globally.^20^ Since obstetric, fistula, and family planning surgical services comprise important surgical interventions in LMICs,^21^ prioritizing learning topics to pursue within these technical areas is a valuable complement to other existing agendas.

We sought to develop a learning agenda, in the context of this project, to identify and prioritize topics in need of exploration across the project’s 4 key technical areas: surgical obstetric care, family planning (long-acting and permanent methods), fistula prevention and treatment, and cross-cutting safe surgery. This learning agenda was developed using a prioritization exercise, in which learning topics were identified and subject matter experts were convened to rate and rank learning topics.

## METHODS

A variety of methods can be used to establish priorities in health research.^22^ Among these include the Delphi method, the Child Health and Nutrition Research Initiative (CHNRI) method, and consultation processes.^22^ The Delphi method involves convening a panel of experts to first solicit topics in an open-ended questionnaire followed by a series of structured questionnaires to rate and rank topics across multiple rounds. Consensus is established by presenting findings back to the expert group and asking them to adjust their ratings over multiple rounds. Common shortcomings of the Delphi method include replicability difficulties and potential for participants with strong opinions or those managing the process to influence participants’ ratings. The CHNRI method also involves convening an expert panel, soliciting research topics from the panel, and asking the panel to score topics according to 5 criteria: answerability, equity, impact on burden, deliverability, and effectiveness.^22^^,^^23^ The CHNRI method relies on the quantitative analysis of scoring data to generate a “collective” result, approximating consensus. Consultation methods involve combinations of focus group discussions and key informant interviews, although consultations reported in the literature tend to provide fewer details on their conduct.^22^

Efforts seeking to establish research priorities in family planning and maternal and newborn health have adopted and modified a variety of approaches. For example, the CHNRI method was used by WHO to establish a global research agenda for family planning,^24^ by the WHO African Region to prioritize SRHR topics,^16^ and by an expert panel convened by WHO to prioritize topics in maternal and perinatal health.^25^ A modified Delphi method was used by a group seeking to establish priorities in respectful newborn care.^20^ Consultation-based methods were utilized by WHO to establish priorities in SRHR for young adolescents,^26^ as well as in efforts to establish research and learning agendas for family planning in 6 countries.^27^ Hybrid approaches involving questionnaires and discussions attempt to take advantage of quantitative measurements from Delphi and CHNRI-based methods while also accounting for potential nuances uncovered through discussion. A hybrid approach was used to develop and validate a quality measurement index for facility-based labor.^28^ Similarly, the USAID-funded Fistula Care *Plus* project adopted a consultation-based method but utilized a questionnaire with scoring criteria (feasibility, potential for impact, saturation, and technical importance) in order establish a quantitative basis for prioritization prior to consensus-building through consultation.^11^

### Consultation Design

In order to prioritize topics for a learning agenda for surgical obstetrics and family planning programming, we solicited the opinion of an expert group through a 2-stage rating and ranking consultation process, adapting and modifying the CHNRI and consultation-based approaches. Specifically, we adapted an approach from a similar research prioritization consultation deployed by the USAID-funded Fistula Care *Plus* project described above.^11^ We modified this hybrid process for the COVID-19 context by holding 2 virtual consultations 1 month apart, with an online rating survey held in between, allowing us to generate research priorities rapidly. This was especially important to us because we intended to use the agenda to identify evidence-generation activities to pursue in order to advance learning in surgical obstetrics and family planning over the life of the project.

We engaged the expert group in a virtual consultation meeting in January 2022 to introduce potential learning topics and rating criteria. We then deployed a survey to the expert group to rate topics and held a second virtual consultation meeting at the end of February 2022 to rank topics by technical area (Figure).

**FIGURE:**
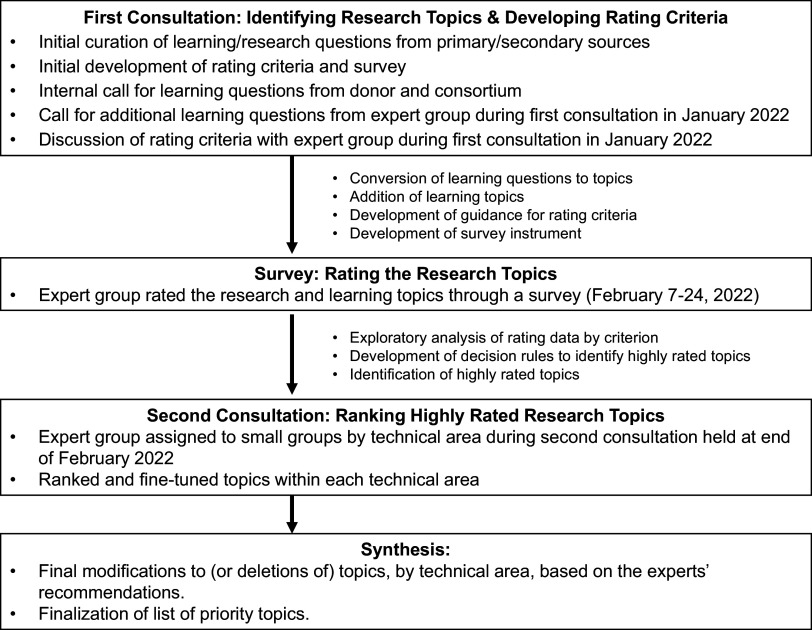
Expert Consultation Process Flow

### Expert Group

The participants engaging in this consultation were identified purposively for their expertise in at least one of the project’s 4 technical areas in the LMIC context: safe surgery, obstetrics, family planning, and fistula prevention and treatment. We sought and obtained input from experts from academia, implementing partner organizations/international NGOs, the donor community, and regional professional associations (Box 1). Project implementation encompassed global and country-specific activity, and consultation participants were primarily identified from organizations at the global and regional levels. As such, women and families were not engaged in this consultation. Global implementing partner organizations work with local institutions, including governments, to build demand for and capacity to provide high-quality health services; while this does not include direct service provision, it often includes research. Participants were invited to participate in the consultations in December 2021, in advance of the first meeting held at the end of January 2022.

BOX 1 Institutional Affiliations of Expert Group Participants

- Aga Khan University Medical College, East Africa
- Bill & Melinda Gates Foundation
- EngenderHealth
- G4 Alliance
- IntraHealth International
- Jhpiego
- Johns Hopkins University Center for Communication Programs
- London School of Hygiene and Tropical Medicine
- Population Council
- Population Reference Bureau
- Program in Global Surgery and Social Change, Harvard Medical School
- United Nations Population Fund
- United States Agency for International Development
- University College London
- University Gamal Abdel Nasser, Conakry, Guinea
- University of Alberta
- University of California, San Francisco
- University of Ibadan, Center for Population and Reproductive Health
- University of Minnesota
- University of Zimbabwe, College of Surgeons of East, Central and Southern Africa
- World Health Organization

### Identifying Research Topics and Developing Rating Criteria

A mix of primary and secondary sources was used to identify research and learning questions, including a survey of attendees of the USAID Fistula Care *Plus* End of Project Learning Event,^29^ the WHO African Region Research Priorities on Sexual and Reproductive Health and Rights,^16^ the technical application for MOMENTUM Safe Surgery in Family Planning and Obstetrics award, and a call for learning topics from core consortium partners and USAID’s Office of Maternal and Child Health and Nutrition and Office of Population and Reproductive Health. Following the initial collection of learning and research topics/questions, topics were edited to be phrased in the form of a question and all research/learning questions were categorized into one of the 4 technical areas of interest mentioned earlier.

The research and learning questions were presented to the expert group during the first consultation meeting held in January 2022. Participants were asked to discuss 2 questions: (1) “Are there glaring gaps—do additional pressing learning questions immediately come to mind?” and (2) “Are there topics with similar questions that need to [be] combined/grouped so that a topic of great interest doesn’t end up rated/ranked lower because of a ‘split vote’?” Participant feedback indicated that the questions were closed-ended in nature, only addressing the “what,” and neglected to understand the “how” or “why” (i.e., pathways to impact). As a result of these discussions, the research questions that we anticipated rating were ultimately converted back into to broader learning topics, along with other additions and deletions made in response to expert group comments.

Rating criteria were adapted from a past research prioritization exercise,^11^ and included feasibility, technical importance, unsaturated topic, and potential for program impact. Ratings followed a 5-point scale, with 1 being the lowest and 5 being the highest (e.g., a rating of “1” for a topic on feasibility indicated very limited feasibility and a rating of “5” for a topic on technical importance indicated a topic of very high importance). Participants in the first consultation meeting were asked to reflect on the criteria categories through the discussion question: “Are there other crucial criteria (other than feasibility, technical importance, lack of saturation, and potential for program impact) that you would recommend be included in the rating survey?” Based on the discussion, the criteria definitions were revised to also account for alignment with country priorities or will/commitment to pursue the topic and ability for a topic to be applied globally (Table 1).

**TABLE 1.** Final Rating Criteria and Definitions Used by the Expert Consultation

Criterion	Definition
Feasibility	Can be addressed with ethical research/learning Can be answered within the resources and time frame available
Technical importance	Has notable gaps in evidence from LMIC settings Is of interest, debate, and discussion among technical peers and leaders in the field Has the potential for a large effect on reproductive, maternal, newborn, child, and adolescent health outcomes and/or the wellbeing of women and newborns
Unsaturated topic	Extensive, quality research/learning relevant to LMIC settings is not already being conducted Avoids redundancy with other projects’ learning agendas (where cross-project learning would not be useful/efficient)
Potential for program impact	Research/learning can result in effective and sustainable interventions/programs Aligns with global and national priorities and commitments Research/learning can clearly lead to action and change how services or programs are delivered in a short time frame Research/learning has potential to generate guidance for large-scale implementation and sustainability through existing systems Prioritizes marginalized groups and/or seeks to reduce inequities in access to services or coverage Relevant to multiple LMICs

Abbreviation: LMICs, low- and middle-income countries.

### Rating the Research Topics

Following the first consultation meeting, the research and learning topics and rating criteria were programmed into a survey (Supplement). The survey instrument was reviewed for usability by 2 members of the expert group. The purpose of this rating survey was to determine which subset of topics rated highest overall. The survey was shared with the expert group and open for responses between February 7, 2022, and February 24, 2022.

Once the survey responses were collected, average ratings were generated for each criterion and each topic, across all participants. For example, an average rating for the “feasibility” of “effective strategies to strengthen key aspects of the safe surgery ecosystem” was calculated. A topic’s overall rating was the average of the average scores of each of the criterion. All criteria were weighted equally in calculation of overall ratings. Topics were then sorted from highest to lowest by overall rating. The distribution of the ratings was analyzed, and decision rules were selected to define which topics to present to the expert group as “highly rated” during the ranking stage. Outliers were analyzed to assess the extent to which the overall rating was influenced by any individual criterion. Given the overlapping nature of the technical areas, all participants were free to rate all the topics.

### Ranking Highly Rated Research Topics

A second virtual consultation session was held at the end of February 2022 to share back which learning and research topics were rated highly and for the expert group to rank these topics for prioritization in a learning agenda.

The expert group was divided into 4 groups, based on their self-reported expertise, among the 4 technical areas of focus mentioned earlier. Each group was shown a list of the top-rated topics meeting the decision rule and proceeded to rank the topics for priority in a learning agenda, taking all the criteria into account holistically. A facilitator managed the discussions in each of the small groups, solicited feedback from all participants, and took notes. In some circumstances, the expert group recommended rewording or merging learning topics.

The small groups also discussed possible specific research and learning questions within the topics, study designs and research methods for investigating the topics, and potential countries in which to conduct research for topics that required primary data collection. The results of these discussions are reported elsewhere.^30^

### Synthesis

Following the second consultation, refinements to topics were made to reflect the recommendations of the expert groups and the list of topics, by technical area, was finalized.

## RESULTS

In total, 39 unique participants of the expert group made contributions across the various stages: 32 attended the first consultation; 33 responded to the survey; and 30 attended the second consultation. Of the 30 attending the second consultation, 29 participated in small groups: 7 in the cross-cutting safe surgery group, 8 in the fistula group, 7 in the family planning group, and 7 in the obstetrics group.

### Overview of Topics

A total of 63 topics were included in the rating exercise: 18 for surgical obstetric care, 20 for fistula prevention and treatment, 8 for family planning, and 17 for cross-cutting safe surgery (Table 2 and Supplement Tables 1-4). These topics were identified following the initial curation of topics from secondary sources (e.g., peer-reviewed literature, project documentation, project-related surveys of experts and partners) and from the consortium of implementing partners within the project, donor, and the expert group. The topic with the highest overall rating was “Effective strategies to strengthen key aspects of the safe surgery ecosystem (e.g., anesthesia supplies, blood and oxygen)” (Supplement Table 4). On average, the cross-cutting safe surgery topics were rated highest, and the fistula prevention and treatment topics were rated the lowest, although the average and the top 25% ratings were closely distributed (Table 2). Specifically, the top 25% ratings for surgical obstetric care, fistula prevention and treatment, and cross-cutting safe surgery were 3.95, 3.99, and 4.07, respectively. Topics rating in the top 25% of their respective technical area were then presented to the expert group for ranking, except for family planning in which the top 5 topics were presented. The top 5 family planning topics were presented for ranking because few family planning topics were curated and applying a similar “top 25%” decision rule would have yielded too few topics for discussion.

**TABLE 2.** Distribution of the Expert Group’s Ratings of Research Topics Across Priority Technical Areas

	Priority Technical Areas			
	Surgical Obstetric Care	Fistula Prevention and Treatment	Family Planning	Cross-Cutting Safe Surgery
Number of topics	18	20	8	17
Minimum rating	3.54	3.25	3.72	3.51
Maximum rating	4.16	4.15	4.09	4.35
Average rating	3.82	3.77	3.85	3.95
Median rating	3.78	3.86	3.82	3.94
Third quartile (Q3) (top 25%)	3.95	3.99	3.89	4.07
Number of topics scoring in top 25%	5	6	2	5
Cutoff/decision rule for subsequent ranking	Q3	Q3	Top 5	Q3

Below, we present the top-rated research topics by priority technical area and the expert group’s discussions during the ranking exercise, followed by the final subset of topics that the expert group prioritized during the ranking exercise.

### Surgical Obstetric Care

#### Rating

Of the 18 surgical obstetric topics rated, 5 rated in the top 25% and were presented back to the expert group for ranking (Table 3). Among these 5 topics, the topic with the highest overall rating was “Using post-discharge/postoperative visits (in person or telehealth) to monitor postoperative morbidity and neonatal outcomes.”

**TABLE 3.** Top Rated Surgical Obstetric Care Research Topics

		Rating				
Topic	n	Feasibility	Technical Importance	Unsaturated Topic	Potential for Impact	Overall
1. Using post-discharge/postoperative visits (in person or telehealth) to monitor postoperative morbidity and neonatal outcomes	28	4.25	4.28	3.86	4.24	4.16
2. Use of tools such as clinical checklists and audits for CD and peripartum hysterectomy to enhance decision-making as part of quality improvement	29	4.55	4.30	3.50	4.17	4.13
3. Intrapartum/midwifery care practices to reduce unnecessary CD	29	4.07	4.34	3.55	4.38	4.09
4. Trial of labor after CD or vaginal birth after CD: LMIC practices, availability/coverage, outcomes	27	4.37	4.30	3.81	4.19	4.01
5. LMIC policies and practices for subsequent pregnancies/deliveries post-CD	28	3.96	4.00	4.00	3.93	3.97

Abbreviations: CD, cesarean delivery; LMIC, low- and middle-income country.

#### Ranking and Synthesis

Consultation participants noted similarities between 2 topics, “Trial of labor after cesarean delivery (CD) (TOLAC) or vaginal birth after CD (VBAC): LMIC practices, availability/coverage, outcomes” and “LMIC policies and practices for subsequent pregnancies/deliveries post-CD,” and elected to retain the former, which was more comprehensive.

Of the topics discussed, “Using post-discharge/postoperative visits (in person or telehealth) to monitor postoperative morbidity and neonatal outcomes” emerged as the highest priority, primarily for its potential for impact. In discussing the importance of post-discharge/postoperative follow-up, participants noted that clinicians know very little about what happens to women and newborns after obstetric surgical procedures and that such knowledge could shed light on the true level of morbidity that a client may be experiencing (e.g., surgical site infections). Participants also discussed the extent to which a follow-up study could be operationalized, highlighting that simple approaches such as phone calls may be feasible and cost-effective. Participants agreed that a meaningful learning outcome would be guidance for practical systems to routinely provide information for quality improvement and for the delivery of follow-up information and care to women and newborns.

Two topics were considered nearly equally important: “Use of tools such as clinical checklists and audits for CD and peripartum hysterectomy to enhance decision-making as part of quality improvement” and “TOLAC or VBAC: LMIC practices, availability/coverage, outcomes.” Participants considered that tools for clinical decision-making could have great potential for impact on quality and outcomes of care within efforts to optimize the cesarean delivery rate and strengthen quality improvement systems. However, given the availability of many tools, learning on proper implementation and consistent use was deemed more important than the development and documentation of a particular tool. The importance of learning on TOLAC and VBAC was emphasized despite the challenges inherent in its learning (e.g., limited provision). Participants also discussed the value of linking research on TOLAC and VBAC to activities related to improving use of the Robson classification system.

Discussants considered learning about intrapartum/midwifery care practices to reduce unnecessary cesarean delivery as important, although at a lower priority than postsurgical follow-up, tools for decision-making, and TOLAC/VBAC. In order to focus this topic on meaningful learning, participants suggested stratifying the topic across 2 sub-topics: (1) specific, effective practices within intrapartum care (e.g., tools to manage labor and ensure birth accompaniment and respectful care), and (2) ways to strengthen midwifery-led models of care that favor less intervention and empower patients, including by understanding why countries trying to implement such models are still struggling to make progress. Moreover, how these issues vary for midwives who work individually versus within a team was noted as an important contextual and learning design factor.

### Fistula Prevention and Treatment

#### Rating

Of the 20 fistula topics rated, 6 rated in the top 25% and were presented back for ranking (Table 4). Among these, the topic with the highest overall rating was “High-impact social and behavior change strategies for community engagement for fistula prevention.”

**TABLE 4.** Top Rated Fistula Prevention and Treatment Research Topics

		Rating				
Topic	n	Feasibility	Technical Importance	Unsaturated Topic	Potential for Impact	Overall
1. High-impact social and behavior change strategies for community engagement for fistula prevention	24	4.17	4.42	3.79	4.21	4.15
2. Coverage of fistula care within basic health care provision funds and social health insurance schemes	23	4.04	4.09	4.3	4.17	4.15
3. Gaps in fistula knowledge/skills in midwifery training	26	4.5	4.35	3.65	3.92	4.11
4. Effective rehabilitation and reintegration interventions for fistula clients	24	3.79	4.33	3.67	4.29	4.02
5. Integration of fistula screening and referral in other health care	23	3.96	4.00	4.09	3.96	4.00
6. Implications of iatrogenic fistula trends for fistula care financing and programming strategies	23	3.96	3.91	4.13	3.96	3.99

#### Ranking and Synthesis

The group initially did not want to rank the 3 highest-rated topics—social and behavior change (SBC), coverage/financing, gaps in knowledge/skills—relative to one another as they were viewed as complementary. Participants noted the importance of learning regarding strengthening provider skills, including among midwives, to prevent fistula through appropriate management or referral of prolonged/obstructed labor, but also noted that such capacity strengthening may not achieve meaningful impact if patients cannot afford or access services. Additionally, there will be no demand for care or behavior change if community members are unaware of service availability, fistula risk factors, and behaviors that can prevent fistula.

When encouraged to rank the topics, “Gaps in fistula knowledge/skills in midwifery training” emerged as the highest priority. However, participants recommended that the topic be expanded to focus on provider competency more broadly. They suggested this topic include exploring where new cases are coming from, technical skills for cesarean delivery and fistula repair, and rehabilitation and reintegration training and care availability. Moreover, participants noted that this topic should address the full continuum, from the community to the facility to the infrastructure of the health system. In addition, participants interpreted the topic of fistula “care” broadly, potentially including prevention and treatment, screening and referral, and rehabilitation services.

Although 3 other topics (rehabilitation and reintegration, integration of screening and referral into other services, and iatrogenic fistula) were considered important, the group concluded that they could be integrated into the remaining topics.

### Family Planning

#### Rating

Of the 8 topics rated by the expert group, 2 topics rated in the top 25%; given this small number, the top 5 were presented back for ranking for parity with the other technical areas (Table 5). Among these 5 topics, the topic with the highest overall rating was “Task sharing for implant removal procedures: feasibility, effectiveness, recommended settings.”

**TABLE 5.** Top Rated Family Planning Research Topics

		Rating				
Topic	**n**	**Feasibility**	**Technical Importance**	**Unsaturated Topic**	**Potential for Impact**	**Overall**
1. Task sharing for implant removal procedures: feasibility, effectiveness, recommended settings	24	4.38	4.13	3.71	4.17	4.09
2. Effectiveness of interventions to increase postabortion contraception uptake and continuance	24	4.08	4.29	3.08	4.46	3.98
3. SBC strategies focusing on men as supportive partners, co-decision makers, and clients of LARCs/PMs	24	4.04	3.96	3.50	3.96	3.86
4. Impact of human-centered designed SBC activities on LARC/PM awareness and uptake	25	3.84	4.20	3.29	4.04	3.84
5. Effective strategies for youth-led organizations to increase capacity to implement evidence-informed interventions to expand access to LARCs/PMs	24	3.58	3.96	3.67	4.00	3.80

Abbreviations: LARCs, long-acting and reversible contraceptives; PMs, permanent methods; SBC, social and behavior change.

#### Ranking and Synthesis

The small group discussed the need for some of the family planning topics to be further expanded. For example, participants felt that the postabortion contraception uptake and continuance topic warranted expansion to include postpartum family planning, and the task sharing for implant removal topic needed to include intrauterine device removal. The group proposed combining the SBC topics due to significant overlap. Although the group recognized the merit of pursuing learning on strategies for youth-led organizations to increase capacity vis-à-vis expanding access to LARCs, the inappropriateness of permanent methods for that age group was discussed, and the topic was narrowed to life course counseling, consent, and/or policies for permanent methods. However, the group discussed other research and implementing partners that are already engaged on this topic and recommended dropping it from consideration in this exercise.

The group also discussed topics that were not highly rated in the survey but considered important nonetheless. Topics related to vasectomy advocacy were noted both due to its underrepresentation in research efforts and its implications for gender equity in family planning. Participants noted that learning related to ensuring matching of supply and demand for vasectomy could be especially meaningful given the method’s historical challenges. The group also discussed the need for learning on tools to support surgical safety and quality within surgical family planning methods, including checklists and monitoring postoperative outcomes.

### Cross-Cutting Safe Surgery

#### Rating

Of the 17 cross-cutting safe surgery topics, 5 were rated by the expert group in the top 25% and were presented back for ranking (Table 6). Among these 5 topics, the topic with the highest overall rating was “Effective strategies to strengthen key aspects of the safe surgery ecosystem (e.g., anesthesia supplies, blood, and oxygen).”

**TABLE 6.** Top Rated Cross-Cutting Safe Surgery Research Topics

		Rating				
Topic	n	Feasibility	Technical Importance	Unsaturated Topic	Potential for Impact	Overall
1. Effective strategies to strengthen key aspects of the safe surgery ecosystem (e.g., anesthesia supplies, blood, and oxygen)	26	4.12	4.65	3.96	4.65	4.35
2. Effective strategies for surgical team capability building in LMICs: teamwork, communication, respectful care	26	3.77	4.35	4.04	4.38	4.13
3. Effective measurement approaches to monitor post-surgical/post-discharge maternal and newborn outcomes in the community	27	3.85	4.15	4.08	4.30	4.09
4. Integration of mental health care skills (e.g., mental health first aid) into fistula/MH/FP provider training	27	4.00	4.07	4.15	4.15	4.09
5. Client definitions of respectful surgical care	25	4.32	3.92	4.12	3.92	4.07

Abbreviations: FP, family planning; LMICs, low- and middle-income countries; MH, maternal health.

#### Ranking and Synthesis

Many of the participants thought that all the cross-cutting topics were important and agreed with their inclusion in the ranking process. However, participants felt that ranking the topics was difficult and contextual; priorities may differ across geography, for example. Additionally, some participants thought that achieving impact on the respectful care topic could be challenging as respectful maternity care more broadly has been incorporated into many policies and procedures already but is still often poorly operationalized in routine maternal care.

Participants agreed that evidence on post-discharge maternal and newborn outcomes at the community level is limited and that learning on these outcomes would address gaps in the quality of care and service delivery processes. They emphasized that the post-discharge period should be a critical part of overall quality improvement initiatives.

### Summary of Prioritized Topics

Following one round of rating and one round of ranking, a total of 15 topics were prioritized across the 4 technical areas explored in this consultation (Table 7).

**TABLE 7.** Research and Learning Topics Ranked as Highest Priority by the Expert Consultation Group

Technical Area	Topics
Surgical Obstetric Care	Use of post-discharge/postoperative visits (in person or telehealth) to monitor postoperative morbidity and neonatal outcomes Use of tools, such as clinical checklists and audits for CD and peripartum hysterectomy, to enhance decision-making as part of quality improvement TOLAC or VBAC: LMIC practices, availability/coverage, and outcomes Intrapartum/midwifery care practices to reduce unnecessary CD, specifically: (1) effective practices within intrapartum care (e.g., tools to manage labor and ensure birth accompaniment and respectful care) and (2) ways to strengthen midwifery-led models of care that favor less intervention and empower patients, including by understanding barriers to progress
Fistula Prevention and Treatment	Gaps in the knowledge/skills required for provision of holistic fistula care, including midwifery/obstetric care, surgical and nonsurgical fistula repair, and rehabilitation High-impact SBC strategies for community engagement for fistula prevention and care Coverage of fistula care within basic healthcare provision funds and social health insurance schemes, including prevention, treatment, screening and referral, and rehabilitation care
Family Planning	Task sharing for LARC removal procedures: feasibility, effectiveness, and recommended settings Effectiveness of interventions to increase voluntary postabortion and postpartum contraception—including LARCs and PMs—uptake and continuance LARC/PM SBC strategies, including impact of human-centered design activities to strengthen awareness and uptake, and strategies focusing on men as supportive partners, co-decision-makers, and clients
Cross-Cutting Safe Surgery	Effective measurement approaches to monitor postsurgical/post-discharge maternal and newborn outcomes in the community Effective strategies to strengthen key aspects of the safe surgery ecosystem (e.g., anesthesia supplies, blood, and oxygen) Effective strategies for surgical team capability building in LMICs, including teamwork, communication, and respectful care Client definitions of respectful surgical care Integration of mental healthcare skills (e.g., mental health first aid) into fistula, MH, and FP provider training to support provision of mental health assessments of and care to patients

Abbreviations: CD, cesarean delivery; FP, family planning; MH, maternal health; SBC, social and behavior change; LARC, long-acting and reversible contraceptives; LMIC, low- and middle-income country; PMs, permanent methods; TOLAC, trial of labor after cesarean delivery; VBAC, vaginal birth after cesarean delivery.

## DISCUSSION

An expert consultation, consisting of open discussions, a rating survey, and a ranking exercise, yielded a core set of actionable research and learning topics across 4 technical areas in reproductive and maternal health surgery: surgical obstetric care, fistula prevention and treatment, family planning, and cross-cutting safe surgery. Although the topics were grouped into 4 distinct categories, this exercise demonstrated the interdependence of topics in safe surgery across technical areas. The prioritization of topics on TOLAC/VBAC and intrapartum/midwifery practices to reduce unnecessary cesarean delivery not only align with WHO recommendations on performing cesarean delivery only when medically indicated^31^ but are also linked to the prevention of iatrogenic fistulas from cesarean delivery complications. SBC strategies were recognized as important for both fistula prevention and treatment and surgical family planning. Many topics touched on the perioperative period (capacity building through teamwork, use of tools for decision-making as part of quality improvement, blood/anesthesia/oxygen, task sharing for LARC removal procedures). Two topics, one each in surgical obstetric care and cross-cutting safe surgery, specifically called out the postoperative period as highly neglected in both evidence and practice. Several domains of respectful maternity care are represented in the prioritized topics across the 4 technical areas.^32^

### Strengths and Limitations

This process adapted an approach and criteria previously developed and applied by a comparable global project to generate a focused priority learning agenda and develop a research portfolio targeting the identified priorities.^11^ The experience and strong engagement of the expert group, comprehensive rating criteria, and iterative nature of the process represent strengths of this consultation. The group of international experts held expertise spanning research and evaluation, clinical practice, and development assistance and implementation across the 4 focus technical areas. The rating criteria enabled the identification of topics where need for further investigation is greatest yet whose investigation could be done practically in the LMIC context. Moreover, the criteria definitions gained additional precision from discussions with the expert group; for example, recommendations resulted in sustainability being included in the criteria definition of potential for impact. The use of a virtual format enabled greater representation of LMIC participants and avoided travel-related emissions. The process enabled expeditious prioritization, enabling the project to focus on pursuing research activities and generating evidence.

However, the approach utilized also featured several limitations. First, as implemented, the representation of the expert group was limited with respect to affiliation. Representation was largely from international organizations and 4 African universities; no Ministry of Health officials participated, nor did women, families, or communities. Second, we did not collect data on gender, geographic location, or occupation (including experience performing focal surgical procedures). Third, although 39 individuals engaged in the rating survey, some attrition was observed (e.g., only 23 responses to the fistula topics), and fewer than 10 people engaged in each of the technical area-specific small group discussions. Fourth, the family planning topics emerging from this process (as designed and implemented) did not generate priorities on voluntary surgical contraceptive methods. It is possible that any of these limitations, including the small group composition, could have introduced bias, especially at the rating stage where small changes in rating could have resulted in any given topic being included or excluded from discussion during the ranking stage. We acknowledge that the composition of topics (both included initially and prioritized) could have been different if any of these design factors were altered (e.g., foregoing a discussion group-based model in favor of a survey-based model that could have accommodated many more participants).

These findings should be interpreted, therefore, as research priorities for a project that was working in LMICs, not an agenda comprehensively applying to all LMICs. As the surgical obstetrics and family planning evidence base continues to grow, we also acknowledge that new lines of inquiry will emerge and that research topics will need to be re-prioritized. Several lessons from this process can inform future prioritization efforts (Box 2).

BOX 2 Lessons Learned for Future Research and Learning Prioritization Efforts

- Time-limited projects seeking to generate their research and learning agendas through prioritization methods must balance the time to generate consensus-based priorities with the time to conduct studies to generate evidence.
- Consultation-based methods can generate rich and contextualized insights, which is helpful for developing research priorities in broad, interdisciplinary spaces (such as safe surgery). However, such methods are inherently limited in the number of participants they can accommodate.
- Developing research priorities that comprehensively apply to all LMICs will require methods that can engage a wider array of actors (e.g., Ministry of Health officials, civil society organizations, academics based in LMICs, patients). Survey-based consensus-building methods (e.g., CHNRI) may be more appropriate for this objective.
- Future prioritization processes should consider gathering participant data on occupation (including experience performing surgery, by procedure), gender, and geographic location to determine whether the research priorities generated reflect the priorities of key subgroups.

We believe that the deficit of topics on voluntary surgical contraceptive methods reflects an overall lack of attention paid to permanent family planning methods. That said, we intentionally set the scope of the prioritization to cover LARCs and permanent methods both because these were the project’s technical areas of focus and because they are procedure-based methods using surgical instruments. However, the topic we included in the initial list on trends and enabling factors in global vasectomy availability and uptake was not rated highly enough to warrant discussion during the ranking stage. This could have been because the expert group was multidisciplinary in nature and was mandated with approximating priorities in the *overall* landscape of surgical obstetrics and family planning. Had the rating process been restricted to participants exclusively involved in the family planning community of practice, it is possible that this topic could have been rated more highly. It is also important to note that a recently published review sheds some light on global vasectomy trends and enabling factors in uptake, as well as persistent barriers to this method receiving advocacy and attention,^33^ and that voluntary surgical contraceptive programming can be studied through the lens of the cross-cutting safe surgery topics prioritized through this process. We nevertheless acknowledge that our approaches could have been adjusted to increase the curation of topics relating to vasectomy and tubal ligation.

### Implications for Research and Practice

It is impossible to avert a significant proportion of maternal morbidity and mortality in LMICs without access to high-quality surgical services delivered to the right patient at the right time. In many parts of the world, however, access to surgery is limited,^3^ referral systems for emergency obstetric conditions remain weak,^34^ preventable perioperative complications persist,^6^^,^^35^ and postoperative care needs reinforcement.^36^ From a human resources for health standpoint, increasing the density of surgeons, obstetricians, and anesthesiologists is correlated with a decline in the maternal mortality ratio, especially in settings with less than 20 specialists per 100,000 population.^2^^,^^37^ Where it is not possible to easily scale up the number of specialists, surgical task sharing to nonspecialist physicians and nonphysician clinicians has been implemented for a variety of procedures in many settings.^38^^,^^39^ In parallel, the number of cesarean deliveries, already the most frequently performed surgical procedure worldwide, has increased in recent years, and is expected to continue increasing.^40^ Therefore, strengthening the provision of safe essential surgery in settings with fragile health systems through obstetric, fistula, and family planning programming can both prevent maternal and newborn morbidity and mortality and serve as a framework for strengthening the entire surgical ecosystem and the ensemble of surgical services provided by health facilities.

This learning agenda contributes to efforts at reducing maternal morbidity and mortality through programmatic and clinical learning across the safe surgery ecosystem, which encompasses processes involved in seeking, reaching, and receiving quality surgical care as well as the linkages after surgical care. High-impact SBC strategies for LARCs/permanent methods and fistula prevention and care, TOLAC/VBAC, and enhancing intrapartum/midwifery care practices to reduce unnecessary cesarean delivery represent learning priorities that aim to prevent complications necessitating surgical care or improve women’s seeking of surgical care when needed. Several topics explore the provision of surgical care, including strengthening the perioperative environment (e.g., anesthesia supplies, blood, oxygen), surgical team capacity building, respectful surgical care, tools for strengthening decision-making, and task sharing for LARC removal procedures. Lastly, several prioritized topics touch upon strengthening continuity of care after surgery, including the use of post-discharge visits, monitoring of postoperative outcomes, integration of mental health care skills in surgical procedures, and gaps in the provision of holistic fistula care. Referral for surgical and emergency obstetric and newborn care is underrepresented in this agenda; however, a significant amount of research has been done on this topic and a recently published systematic review of referral interventions for obstetric emergencies proposes a logic model for “studying, understanding and improving” such processes.^34^

Because the research priorities emerging from this process are programmatic and clinical in nature, this agenda neglects topics that may be a few steps removed from service delivery but may, nevertheless, be crucial for strengthening surgical systems in the long term. For example, this agenda does not cover policy questions, such as the generation of political support for safe surgery,^41^ or effective strategies for developing and implementing National Surgical, Obstetric, and Anesthesia plans in more LMICs. This outcome, though unintended, may be the result of strong representation of implementing partners in developing this agenda and because the rating criteria, specifically feasibility and potential for impact, may have contributed to prioritization of topics whose pursuit could achieve the most impact with the fewest resources. However, this agenda is not intended to stand alone, but rather to complement other existing learning agendas at the global and national levels, including on maternal health, family planning, safe surgery in LMICs (e.g., general, pediatrics, orthopedics), and health systems and policy research.

### Implementation of the Research and Learning Agenda

The MOMENTUM Safe Surgery in Family Planning and Obstetrics project used this learning agenda to guide its research and learning strategy, including a series of activities involving special studies and targeted literature reviews (Table 8). Whereas some of these activities were initiated at the global level, others were launched in response to research priorities established by national and local stakeholders. Examples of the latter include the measurement of postsurgical complications before and after the implementation of a peripartum surgery diploma in Mali and the Robson classification study in Senegal. Indeed, one of the benefits of the broad nature of many of the learning agenda topics is that they offer opportunities for alignment with research priorities established at the national and subnational levels. In addition, we also supported Ministries of Health in generating evidence for topics that were local priorities but that fell outside the learning agenda’s scope.

**TABLE 8.** Illustrative Contributions of MOMENTUM Safe Surgery in Family Planning and Obstetrics Project Activities to Advancing Priority Learning Agenda Topics

**Learning Agenda Topic**	**Project Learning Activity**
Use of tools, such as clinical checklists and audits for cesarean delivery and peripartum hysterectomy, to enhance decision-making as part of quality improvement	Safe cesarean checklist: review of the literature, co-design, and pilot testing Cesarean delivery audit tool development: review of literature for existing tools, provider survey on audit practices and procedures, and design/piloting of consolidated audit tool Cross-sectional study measuring cesarean delivery complications before and after the implementation of a peripartum surgery diploma program in Mali Descriptive study on sociodemographic and clinical factors of patient populations undergoing cesarean delivery in Senegal (Robson classification) Analysis of data captured from the piloting of a cesarean delivery audit mobile application in India
Task sharing for LARC removal procedures: feasibility, effectiveness, and recommended setting	Review of training guidelines, regulatory frameworks, and scopes of work to assess the extent to which WHO task sharing guidelines on LARCs and PMs are being implemented in 7 countries^42^ Survey on provider perspectives on implant and IUD removal
Use of post-discharge/postoperative visits (in-person or telehealth) to monitor postoperative morbidity and neonatal outcomes	Prospective cohort study testing the feasibility of post-discharge phone interviews as a method of monitoring post-discharge outcomes in DRC
Effective strategies to strengthen key aspects of the safe surgery ecosystem (e.g., anesthesia supplies, blood, and oxygen)	Qualitative study assessing extent to which safe surgery core teams are perceived as effective in driving quality improvement in surgical care in Rwanda Contribution/influence of anesthesia clinical mentorship on mitigating anesthesia complications in Mali and Rwanda

Abbreviations: DRC, Democratic Republic of the Congo; IUD, intrauterine device; LARCs, long-acting reversible contraceptives; PMs, permanent methods.

### Opportunities for the Broader Reproductive and Maternal Health Community

Although this agenda was primarily developed for a global project working in LMICs, a time-bound, donor-funded project can only undertake a limited level of learning on such a diverse set of priorities. Evidence on the topics prioritized in this collaborative learning agenda can be advanced by domestic and donor agencies, research institutions, and implementing partners supporting maternal and reproductive health service strengthening.

Indeed, priorities at the national and sub-national levels may not necessarily align with priorities at the global level. As such, research prioritization and evidence generation efforts at the national and subnational levels ought to be led by Ministry of Health officials as well as their partners (e.g., academia, implementing partners, civil society, the private sector, and community-based organizations). As mentioned above, this agenda was primarily developed as a project’s learning agenda and does not intend to substitute national/subnational efforts, but rather to complement them. The topics prioritized in this agenda may, however, support national and subnational stakeholders in identifying potential lines of inquiry to prioritize or interventions to study.

Although the vertical nature of most traditional development assistance for health presents a challenge for learning resource mobilization given the integrated or horizontal nature of safe surgery systems,^41^ large-scale donor-funded health programs may want to consider adopting these topics when developing their evidence generation strategies. Stratifying this learning exercise by obstetrics, fistula, family planning, and cross-cutting safe surgery topics can enable implementing partners to consider topics that align with their programmatic mandates, whether vertical or integrated. A maternal health program may, for example, consider investigating the obstetrics topics. As basic and essential surgery is increasingly being recognized as a key component of primary health care,^43^ an integrated primary health care program working across several technical areas may consider designing implementation research studies with these topics in mind. Many of the cross-cutting topics identified in this agenda can be studied even through the lens of vertical interventions. For example, client definitions of respectful care or strategies for surgical team capacity building can be studied in the context of cesarean deliveries, permanent family planning methods, and/or fistula repair.

That said, the prioritization of topics in this agenda that inherently build on and overlap with each other further emphasizes the integrated nature of the common ecosystem that support surgeries across different technical areas (e.g., cross-cadre surgical teams, safe blood and oxygen systems, and monitoring of postsurgical outcomes) and suggest opportunities for funding research and programming that can be conducted and applied to a range of services. Given the relative newness of global safe surgery as a global public health priority, we view the development of this agenda as a starting point, not an end point, for a dialogue across communities of practices working at different levels (e.g., academics, funders, and implementers).

## CONCLUSION

Through an expert consultation, research and learning topics were identified and prioritized across 4 technical areas of a safe surgery project that comprise important interventions in maternal health and family planning that are growing in volume and whose availability, access, quality, and safety need to be strengthened for optimal impact and use of resources. These topics may warrant consideration for exploration in the research and learning efforts of national, regional, and global maternal health and family planning initiatives, including via targeted investment of domestic and donor resources.

## Notes

**Peer Reviewed**

**First Published Online:** December 15, 2025.
